# Genetic variants in lncRNA *H19* are associated with the risk of oral squamous cell carcinoma in a Chinese population

**DOI:** 10.18632/oncotarget.23673

**Published:** 2018-01-02

**Authors:** Zhiyao Yuan, Yang Yu, Bo Zhang, Limin Miao, Lihua Wang, Keke Zhao, Yefeng Ji, Ruixia Wang, Hongxia Ma, Ning Chen, Hua Yuan

**Affiliations:** ^1^ Jiangsu Key Laboratory of Oral Diseases, Nanjing Medical University, Nanjing, China; ^2^ Department of Oral and Maxillofacial Surgery, Affiliated Hospital of Stomatology, Nanjing Medical University, Nanjing, China; ^3^ Department of Epidemiology and Biostatistics, Jiangsu Key Laboratory of Cancer Biomarkers, Prevention and Treatment, Cancer Center, School of Public Health, Nanjing Medical University, Nanjing, China

**Keywords:** lncRNAs, H19, oral squamous cell carcinoma, genetic variants

## Abstract

To evaluate whether the genetic variants in *H19* influence the risk of oral squamous cell carcinoma (OSCC) in a Chinese population, a case-control study was conducted to analyze four functional single nucleotide polymorphisms (SNPs) in *H19*. The cohort comprised of 444 OSCC cases and 984 healthy controls, and the study further evaluated the biological effect by bioinformatics prediction and functional experiments. Two SNPs, rs217727 and rs2839701, were found to be associated with the risk of OSCC [rs217727: odds ratio (OR) = 1.32, 95% confidence interval (CI) = 1.11–1.58, *P* = 0.002; rs2839701: OR = 1.23, 95% CI = 1.04–1.46, *P* = 0.019].Bioinformatics predicted that rs2839701 C>G might alter the secondary structure of *H19*. In addition, rs2839701 C>G inhibited the transcription activity and was correlated with the decreased expression of downstream gene *MRPL23-AS1* that was downregulated in OSCC. The current results suggested that the SNPs in H19 may play a major role in genetic susceptibility to OSCC.

## INTRODUCTION

Oral squamous cell carcinoma (OSCC) is a severe disease worldwide, with high morbidity and 5-year mortality of about 50% [1]. In China, an estimated 48,100 incidents and 22,100 death cases due to oral and pharynx cancer were reported in 2015 [2]. Smoking and alcohol consumption are the main environmental factors affecting the development of OSCC [3], although genetic variants also play vital roles in oral carcinogenesis [4]. Hitherto, two genome-wide association studies (GWAS) have revealed several oral cancer susceptibility loci [5, 6]; however, there is yet much of heritability unexplained. Therefore, the remaining unidentified heritability necessitates further investigation.

Long non-coding RNAs (lncRNAs) are defined as RNAs > 200 nucleotides in length and those without encoding proteins; these could serve as transcriptional and post-transcriptional regulators in carcinogenesis [7]. *H19* is one of the lncRNAs that participates in the development and progression of malignancies [8]. Several studies have shown aberrant expression of *H19* in multiple cancers including squamous cell carcinoma of head and neck [9]. *H19* may participate in tumorigenesis and progression by acting as competing endogenous RNA (ceRNA) [10] or the precursor of microRNA [11]. For example, *H19* could promote the development of gallbladder cancer by competitively binding miR-342-3p to regulate the expression of FOXM1 [12]. Micro RNA (miRNA)-675, encoded by exon1 of *H19*, enhances the proliferation and invasion of gastric cancer via tumor suppressor *RUNX1* [13].

Single-nucleotide polymorphism (SNP) is a kind of genetic variation that might influence gene expression and mRNA conformation, thereby affecting the function of the gene and disease phenotype [14]. With the development of bioinformatics, unique datasets and prediction software have provided exhaustive data for gene function and expression influenced by functional SNPs. For example, the Genotype-Tissue Expression (GTEx) Project has established a tissue bank for studying the association of genetic variations in gene expression in various tissues or organs of a human body, thereby providing evidence for the expression quantitative trait loci (eQTL) [15, 16]. The Encyclopedia of DNA Elements Consortium (ENCODE) provides information of functional regulatory elements in the whole human genome [17]. Recently, SNPs in lncRNAs have gained increasing attention for their physiological regulatory functions. The genetic variants of *H19* have been identified to be associated with the susceptibility to breast cancer [18], bladder cancer [19], gastric cancer [20], and colorectal cancer [21]. However, there is no study addressing the relationship between genetic variations of *H19* and OSCC risk in Chinese population.

Therefore, we hypothesized that functional SNPs of *H19* might influence the physiological function of *H19* or the expression of cancer-related genes, thereby contributing to the development of OSCC. To confirm this hypothesis, a case–control study was conducted to evaluate the associations between functional SNPs of *H19* and OSCC risk using a cohort of 444 cases and 984 controls, and further evaluating the physiological effect by bioinformatics prediction and functional experiments.

## RESULTS

### General characteristics of participants

The distributions of selected variables in the study subjects are characterized in Table 1. No significant difference was observed between OSCC cases and controls (*P* = 0.433 for age; *P* = 0.242 for gender). However, the number of smokers and drinkers in cases was more than that in controls (smoking status: 40.32 vs. 34.86%, *P =* 0.047; drinking status: 41.44 *vs.* 30.89%, *P <* 0.001).

**Table 1:** General characteristics of participants

Variables	Case	Control	*P*^a^
	*N* (%)	*N* (%)	
**Participants**	444 (100)	984 (100)	
Age			0.433
<60	203 (44.19)	428 (43.50)	
≥60	241 (54.28)	556 (56.50)	
Gender			0.242
Females	195 (43.92)	465 (47.26)	
Males	249 (56.08)	519 (52.74)	
Smoking status			0.047
Never	265 (59.68)	641 (65.14)	
Ever	179 (40.32)	343 (34.86)	
Drinking status			<0.001
Never	260 (58.56)	680 (69.11)	
Ever	184 (41.44)	304 (30.89)	

^a^Two-sided χ^2^ test was used.

### Associations of selected SNPs with OSCC risk

As shown in Table 2, the preliminary information of the four selected SNPs was listed, and the genotype distributions among controls conformed to the Hardy–Weinberg equilibrium (HWE) (*P* > 0.05). Among these four SNPs, rs217727 and rs2839701 were found to be related to the risk of OSCC in the additive model after adjusting for age, gender, smoking, and alcohol drinking status. The genotype distributions of rs217727 and rs2839701 and the association with the risk of OSCC were shown in Table 3. Subjects with rs217727 TT genotype were more susceptible to the development of OSCC as compared to those carrying CC (OR = 1.89, 95% CI: 1.27–2.82), while the rs2839701 GG carriers had a 1.64-fold elevated risk of developing OSCC as compared to individuals with the CC genotype (95% CI = 1.10–2.43). Further combined analysis revealed that with an increased number of risk alleles, the risk of OSCC elevated significantly (*P*_trend_ < 0.001). The rs217727T and rs2839701G were speculated as risk alleles based on the primary effect of the individual locus. Subjects carrying “2-3” risk alleles showed a 2.22-fold elevated OSCC risk (95% CI = 1.56–3.16) as compared to the “0” group (Supplementary Table 1).

**Table 2:** Primary information and minor allele frequencies (MAFs) of selected SNPs

SNPs	Gene	Chr	Location	Base change	MAF^a^ in dbSNP database (CHB)	MAF in our controls	HWE^a^	Genotyping rate (%)	*P*^*b*^
rs217727	*H19*	11p15.5	exon	C>T	0.354	0.289	0.163	99.1	**0.002**
rs2839701	*H19*	11p15.5	exon	C>G	0.308	0.281	0.752	100	**0.019**
rs2067051	*H19*	11p15.5	exon	G>A	0.244	0.273	0.630	99.1	0.144
rs2251375	*H19*	11p15.5	intron	C>A	0.458	0.424	0.134	100	0.702

^a^HWE = Hardy-Weinberg equilibrium. MAF = minor allele frequency.

^b^Derived from logistic regression with an adjustment for age, sex, smoking and drinking status inadditive model.

**Table 3:** Genotypes of selected SNPs and OSCC risk in different models

Positive SNPs	Calculation models	Case (%) (*n* = 444)	Control (%) (*n* = 984)	Adjusted OR ^a^(95%CI)	*p*^*a*^
rs217727	CC	186 (41.89)	488 (49.59)	1	
	CT	194 (43.69)	423 (42.99)	1.24 (0.97-1.58)	0.080
	TT	51 (11.49)	73 (7.42)	**1.89 (1.27-2.82)**	**0.002**
	Dominant			**1.34 (1.06-1.68)**	**0.013**
	Recessive			**1.70 (1.16-2.49)**	**0.006**
	Additive			**1.32 (1.11-1.58)**	**0.002**
rs2839701	CC	205 (46.17)	507 (51.52)	1	
	CG	188 (42.34)	402 (40.85)	1.15 (0.91-1.46)	0.248
	GG	51 (11.49)	75 (7.62)	**1.64 (1.10-2.43)**	**0.014**
	Dominant			1.23 (0.98-1.54)	0.075
	Recessive			**1.53 (1.05-2.24)**	**0.027**
	Additive			**1.23 (1.04-1.46)**	**0.019**

^a^ORs and 95% CIs were calculated after adjusting for age, sex, smoking and drinking status by using logistic regression analyses.

### Stratification analysis

We also conducted stratification analyses and found that the risk effect of rs217727 was strong among elderly (age≥ 60-year-old) (OR = 1.35, 95% CI: 1.07–1.70) and nonsmokers (OR = 1.34, 95% CI: 1.07–1.67); in addition, the association between rs2839701 variants and the risk of OSCC was rather profound among the elderly group (adjusted OR = 1.33, 95% CI: 1.06–1.67), females (adjusted OR = 1.47, 95% CI: 1.13–1.90), nonsmokers (adjusted OR = 1.29, 95% CI: 1.03–1.61) and nondrinkers (adjusted OR = 1.25, 95% CI: 1.00–1.56) (Supplementary Table 2). Moreover, no significant heterogeneity was observed in each subgroup (Supplementary Table 3).

### Biological function of rs2839701 on transcription activity

**Figure 1:**
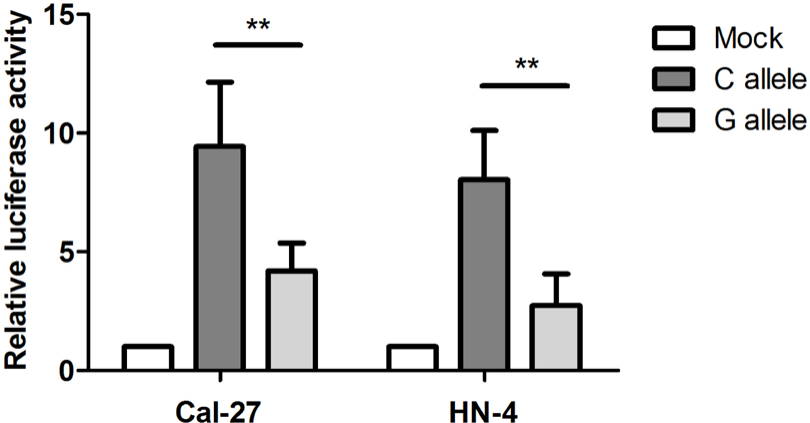
Rs2839701 C>G significantly inhibited transcription activity in OSCC cell lines The results are shown as a ratio of firefly luciferase activity in relation to the control pRL-SV40 renilla luciferase. The mean fold change ± SD for plasmid with different alleles are measured by defining the radio of empty control vector(mock) as 1. ^**^P < 0.001.

Bioinformatics prediction based on SNPinfo (http://snpinfo.niehs.nih.gov/) indicated that rs2839701 might influence the binding of the transcription factors of the two positive SNPs. Therefore, we performed functional experiments to investigate whether rs2839701 affected the transcription activity. Since rs2839701 was localized in the region enriched of the trimethylation of lysine 4 of the H3 (H3K4Me3) histone mark representing promoter activity (data from ENCODE), we evaluated its regulatory role by luciferase reporter assay. Both CAL27 and HN4 cells transfected with vectors containing the risk allele “G” showed a lower normalized luminescence as compared to the C allele, indicating an inhibitory effect of rs2839701 risk “G” allele on the transcription activity (CAL27: 9.43±2.71 *vs.* 4.19 ± 2.18, *P* = 0.004; HN4: 8.03 ± 2.08 *vs.*2.74 ± 1.33, *P* = 0.001, Figure 1).

### *In silico* prediction of secondary structure caused by rs2839701

**Figure 2:**
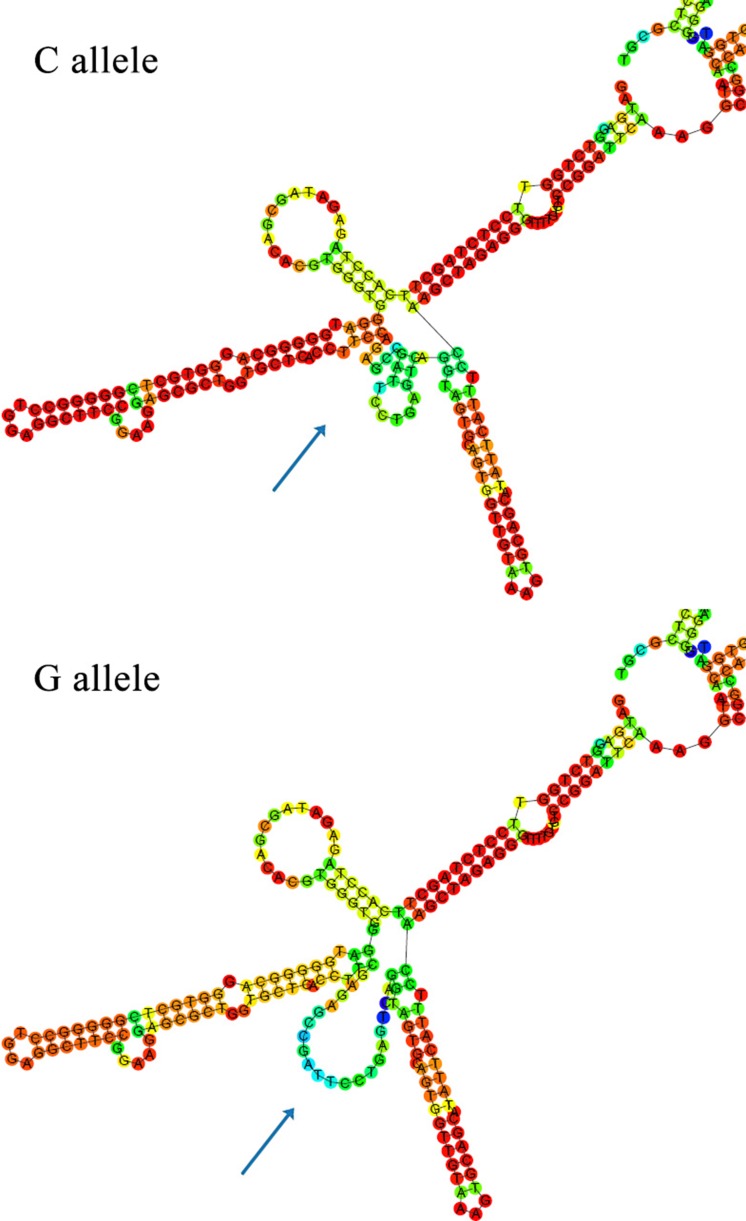
*In silico* prediction of secondary structure of *H19* with RNAfold The arrow indicates alteration in structures caused by rs2839701 C/G variant.

*In silico* analyses performed by RNAfold predicted the influence of rs2839701 on the secondary structure of *H19*. As shown in Figure 2, the secondary structure was remarkably altered with rs2839701 C/G variants.

### Effects of rs2839701 on potential cancer-related gene

**Figure 3:**
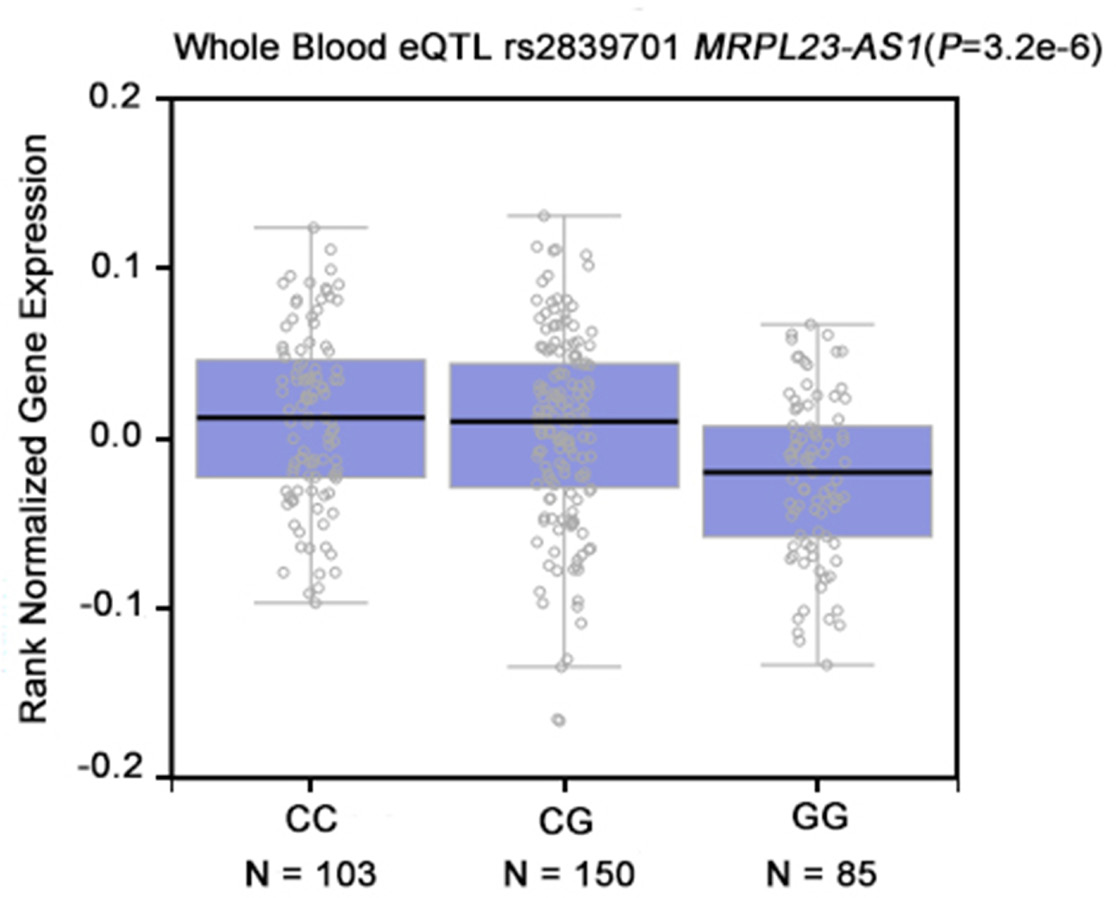
The genotype of rs2839701 was correlated with the expression of *MRPL23-AS1* in whole blood (data from GTEx Portal).

**Figure 4:**
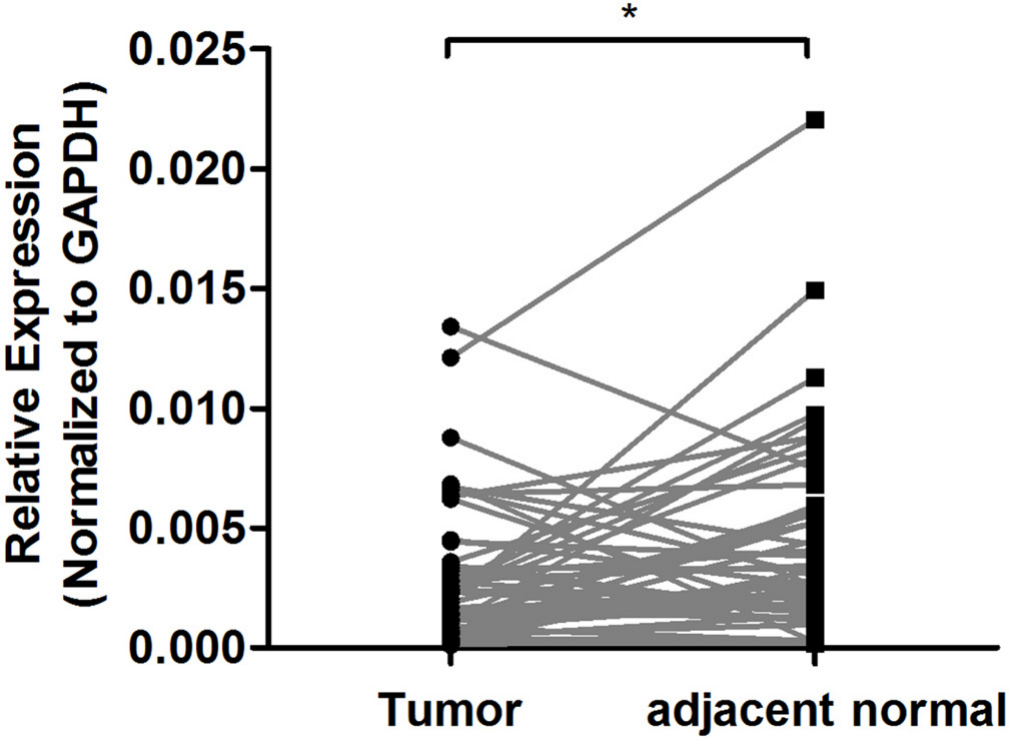
Expression analysis of *MRPL23-AS1* in 51 paired of OSCC tissues and adjacent normal tissues The method of 2^–ΔCT^ was used to calculate relative expression levels normalized by GAPDH.^*^P < 0.05.

To further explore the biological effect of rs2839701, we investigated whether rs2839701 had an allele-specific correlation on the proximal cancer-related genes. The eQTL evidence was based on the public GTEx (http://www.gtexportal.org/home/) database was searched, and rs2839701C>G was found to remarkably correlate with the decreased expression of *MRPL23-AS1* (*P* < 0.001, Figure 3). *MRPL23-AS1* is a noncoding gene located 5.9 kb downstream of rs2839701. Next, we evaluated the expression of *MRPL23-AS1* by RT-PCR in 51 pairs of OSCC tissues and the adjacent normal tissues. Our results showed that the expression level of *MRPL23-AS1* in OSCC tissues was lower than that in normal tissues (*P* = 0.0125, Figure 4).

## DISCUSSION

Long noncoding RNAs act as regulators of several biological processes including carcinogenesis. Emerging evidence has indicated that SNPs of lncRNAs could influence the functions and expression of cancer-related genes, thereby contributing to cancer susceptibility. For example, Du *et al.* [22] identified a gastric cancer susceptibility SNP rs4759314 on lncRNA *HOTAIR* could regulate the expression of *HOTAIR* and the proximal gene *HOXC11*. Herein, a case-control study was conducted to investigate whether the functional SNPs in *H19* were associated with the risk of OSCC. Among the four SNPs, rs2839701 C>G and rs217727 C>T were found to be correlated with increased OSCC risk independently and jointly.

The biological mechanisms underlying disease-associated SNPs have gained increasing attention with respect to molecular epidemiology. For example, Li *et al.* reported that rs3764482 C>T variant was associated with colorectal cancer risk by modulating the TGF-β signaling [23]. rs6695837, localized at 2kb upstream of the *LAMC1* gene, affected the *LAMC1* promoter activity and contributed to colorectal cancer susceptibility [24]. The SNP rs2839701 resides on the exon 4 of *H19*. Secondary structure plays a major role in the function of lncRNAs [25]. We performed *in silico* analyses by RNAfold and found that the secondary structure of *H19* altered with rs2839701 C/G alleles. In addition, based on the ENCODE data, rs2839701 localized in the region exhibiting the promoter activity. The luciferase reporter assay showed an inhibitory effect of rs2839701 risk “G” allele on transcription activity. We hypothesized that the risk SNP rs2839701 had an allele-specific correlation on the nearby genes. Intriguingly, rs2839701 risk “G” allele was significantly associated with low expression levels of 5.9 kb downstream gene *MRPL23-AS1*, which was less expressed in the OSCC tissues. Our results suggested that rs2839701 inhibited the transcriptional activity that might be associated with the decreased expression of a potential tumor suppressor gene *MRPL23-AS1* and raised the risk of OSCC. Furthermore, the SNP rs2839698, with a high linkage disequilibrium (*r*^*2*^ > 0.8) of rs2839701, has been evaluated for the genetic susceptibility to several cancer types. A meta-analysis performed by Chu *et al.* [26] demonstrated that rs2839698 was related to the risk of digestive system cancers, which was in agreement with the results of the current study on rs2839701.

Reportedly, the relationship between rs217727 polymorphism and the susceptibility to cancers in Chinese population have not yet achieved a consensus. The *H19* rs217727 polymorphism has been shown to be associated with the risk of gastric cancer [20], breast cancer [18], and bladder cancer [19]; however, no significant association was observed between rs217727 and colorectal cancer risk [21] in the Chinese population. Lu *et al.* [27]conducted a meta-analysis and suggested that rs217727 polymorphism did not correlate with the susceptibility to cancers in Chinese individuals. Nevertheless, our study showed that rs217727 C>T exhibited a significantly increased risk for the development of OSCC. Therefore, cumulative meta-analysis is warranted in order to obtain definitive results.

Nonetheless, several limitations should be considered in the present study. First, the allele-specific correlation between rs2839701 and *MRPL23-AS1* was based on the GTEx database; however, we did not evaluate this correlation in OSCC samples. Second, we failed to retrieve the predicted transcription factors binding site (TFBS) of rs217727 by SNPinfo; thus, further functional experiments are essential to clarify the biological mechanisms of rs217727 on the development of OSCC. Third, our study was focused on the molecular epidemiology of OSCC development, and the investigation on the potential mechanism of rs2839701 C>G was preliminary. More experimental evidences are needed to verify the predicted data. Final, this one-stage study was conducted with a relatively small sample size, and hence, large-scale studies are imperative to validate our findings.

In conclusion, we identified two SNPs, rs2839701 and rs217727, associated with OSCC susceptibility in a Chinese population. The functional SNP rs2839701 C>G may alter the secondary structure of *H19* and influence the function, which might partially explain the OSCC genetic susceptibility. In addition, rs2839701 genetic variant inhibited the transcriptional activity and was correlated with the decreased expression of downstream gene *MRPL23-AS1* that is downregulated in OSCC. Therefore, our results suggested that the SNPs in *H19* might serve as potential biomarkers of OSCC prediction.

## MATERIALS AND METHODS

### Study population

In the present study, we enrolled 444 OSCC cases and 984 controls. From January 2009 to May 2013, we recruited 444 histopathologically confirmed patients from Jiangsu Stomatological Hospital and Nanjing Medical University First Affiliated Hospital, excluding those with metastatic tumors and received chemotherapy or radiotherapy. Age- (±5 years) and gender-matched controls were chosen randomly from a community-based disease screening project, including >30,000 individuals during the same period, which was carried out in the Jiangsu Province. The participants were interviewed personally by uniformly trained interviewers to obtain general and lifestyle information with respect to age, gender, smoking, and alcohol drinking. Moreover, withdrew a 5 ml venous blood sample from each participant for genomic DNA isolation and genotyping. Every participant signed an informed consent. The present study was approved by the Ethics Committee of Nanjing Medical University.

### SNP selection and genotype identification

The region including 2 kb upstream of the *H19* gene was screened by the HapMap database (version: phase II + III Feb 09, dbSNP b126) in Chinese Han population (CHB), according to the following criteria: *P* for Hardy–Weinberg equilibrium (HWE) ≥0.05; genotyping rate ≥90%; minor allele frequency (MAF) ≥0.05. Thus, 9 SNPs were selected. Then, according to an online function-scoring software (http://www.regulomedb.org/), 7 potentially functional SNPs were included for their RegulomeDB score ≤4. This database was supported by Stanford University. Every variant was graded by its function on the binding of transcription factors and the expression quantitative trait. The higher the score, the lower the function. Lastly, four tagging SNPs were selected by Haploview 4.2 software according to *r*^*2*^ for linkage disequilibrium (LD) <0.8.

Genomic DNA was extracted from peripheral blood leukocytes by proteinase K digestion and phenol-chloroform methods. Sequenom MassARRAY iPLEX platform was used for genotyping the SNPs.

### Luciferase reporter assay

A 207-bp human genomic region (UCSC hg38, chr11: 1995795–1996001), surrounding the functional candidate SNP rs2839701 C and G alleles, was synthesized and inserted into the pGL3-promoter plasmids (Promega), respectively, digested with 5′-*SacI* and 3′-*XhoI*. The constructs were validated by DNA sequencing.

### Construct risk allele (C)

CTAGAGATAGCGACACGTGGGTGGGATGGGGGCAGGGTGCTCGGGGGCCTGGAGGCTTCCGGAAGGAGCGCTGGTGCTCACCTTC**C**AGAGCCGATTCCTGAGTCAGGTAGTGCAGTGGTTGTAAAGTGCAGCATATTCATTTCCAAGCTAGAGGGTTTTGTGTCCGGATTCAAAGGCCCAGGCTTGAGCTGGGTAGCACCATTTCTG

### Construct wild allele (G)

CTAGAGATAGCGACACGTGGGTGGGATGGGGGCAGGGTGCTCGGGGGCCTGGAGGCTTCCGGAAGGAGCGCTGGTGCTCACCTTC**G**AGAGCCGATTCCTGAGTCAGGTAGTGCAGTGGTTGTAAAGTGCAGCATATTCATTTCCAAGCTAGAGGGTTTTGTGTCCGGATTCAAAGGCCCAGGCTTGAGCTGGGTAGCACCATTTCTG.

Cal-27 and HN-4 cells were cultured in a humidified incubator at 37°C with 5% CO_2_. The medium used for cell culture was DMEM containing 10% fetal bovine serum. Cells were seeded in 24-well plates with 5 × 10^4^ cells/well and subsequently transfected with 500 ng luciferase reporter plasmids by Lipofectamine 2000 (Invitrogen) after 24 h. 10 ng of Renilla luciferase pRL-SV40 plasmid (Promega, Wisconsin, USA) was cotransfected per well as an internal control. 48 h post-transfection, the cells were analyzed for the luciferase activities by the Dual-Luciferase Reporter Assay System (Promega). All the reporter assays were conducted at least three times in quintuplicate (five technical replicates each).

### *In silico* prediction of secondary structure change

To investigate whether SNP rs2839701 changed the secondary structure of *H19*, we performed an *in silico* analyses using the online prediction tool, RNAfold (http://nhjy.hzau.edu.cn/kech/swxxx/jakj/dianzi/Bioinf4/miRNA/miRNA1.htm).

### Analysis of *MRPL23-AS1* expression levels in OSCC tissues

Total RNAs were extracted from 51 OSCC specimens, and 51 paired adjacent normal tissues by TRIzol (Invitrogen, CA, USA). The cDNA was synthesized using the PrimeScript RT Master Mix Kit (TaKaRa, Dalian, China). Subsequently, we evaluated the expression levels of *MRPL23-AS1* byreal-time reverse transcription PCR utilizing the SYBR Premix EX Taq Kit (TaKaRa Biotechnology, Japan).The expression level of the gene was normalized to *GAPDH* [24]. The primers used were as follows: *MRPL23-AS1* (forward,5′-GAGGTTCCTGTGGGAGTTGG-3′; reverse, 5′-TCACCCTGTCTTCGCCTGT-3′) and *GAPDH* (forward, 5′-GGGCTGCTTTTAACTCTGGT-3′; reverse, 5′-GCAGGTTTTTCTAGACGG-3′). The amplification program was set as 95°C for 30s, followed by 40 cycles of 95°C for 5s and 60°C for 30s on the ABI 7900 HT real-time PCR system (Applied Biosystems, USA). The method of 2^-ΔCT^ [28] was used to calculate the relative expression levels, and all reactions were performed in triplicate.

### Statistical analysis

The differences in general characteristics between OSCC cases and controls were evaluated by χ^2^ test. The goodness-of-fit χ^2^ test was used to calculate the HWE in the control subjects. Logistic regression estimated the associations between genotypes of selected SNPs and OSCC risk by assessing the odds ratios (ORs) and 95% confidence intervals (CIs). The χ^2^-based *Q*-test was used to compute the heterogeneity between subgroups. *P* < 0.05 was considered as statistically significant. All the statistical analyses were two-sided and conducted with Stata software (version 11.0; Stata Corporation, TX, USA).

## SUPPLEMENTARY MATERIALS TABLES



## Abbreviations

OSCC: oral squamous cell carcinoma
GWAS: genome-wide association study
SNPs: single nucleotide polymorphisms
lncRNA: long non-coding RNA
OR: odds ratio
CI: confidence interval
ENCODE: Encyclopedia of DNA Elements
GTEx: Genotype-Tissue Expression
eQTL: expression quantitative trait loci
HWE: Hardy–Weinberg equilibrium
LD: linkage disequilibrium
H3K4Me3: tri-methylation of lysine 4 of the H3
TFBS: transcription factors binding site
