# Supplementary material for: Genetic variants in lncRNA H19 are associated with the risk of oral squamous cell carcinoma in a Chinese population
Source: Oncotarget. 2018 Jan 2;9(35):23915–22. doi: 10.18632/oncotarget.23673 (PMC5963630; doi:10.18632/oncotarget.23673)
Supplement: Supplementary file 1 [file oncotarget-09-23915-s001.pdf]

Genetic variants in lncRNA *H19* are associated with the risk of oral squamous cell carcinoma in a Chinese population

SUPPLEMENTARY MATERIALS

Supplementary Table 1: Cumulative effect of rs217727 and rs2839701 on the risk of OSCC

No. of risk allele[a]	Case *N* (%)	Control *N* (%)	Adjust OR (95%CI)[b]	*P*[b]
0	53 (12.30)	186 (18.90)	1	
1	178 (41.30)	475 (48.27)	1.36 (0.95-1.93)	0.090
2-3	200 (46.40)	323 (32.83)	**2.22 (1.56-3.16)**	**<0.001**
Trend			**1.53 (1.29-1.81)**	**<0.001**
Binary classification				
0-1	231 (53.60)	661 (67.17)		
2-3	200 (46.40)	323 (32.83)	**1.76 (1.40-2.23)**	**<0.001**

[a]The rs217727T and rs2839701G were assumed as risk alleles based on main effect of individual locus.
[b]Derived from additive model using logistic regression analyses with an adjustment for age, sex, smoking and drinking status.

Supplementary Table 2: Stratified analyses of association between SNPs and OSCC

Variables	rs217727 Case CC/CT/TT	rs217727 Control CC/CT/TT	Adjusted OR[a] (95% CI)	*p*[a]	rs2839701 Case CC/CG/GG	rs2839701 Control CC/CG/GG	Adjusted OR a (95%CI)	*p*[a]
Age, yr								
<60	82/94/20	203/196/29	1.29 (0.98–1.69)	0.066	100/82/21	213/189/26	1.11 (0.85–1.45)	0.444
≥60	104/100/31	285/227/44	**1.35 (1.07–1.70)**	**0.011**	105/106/30	294/213/49	**1.33 (1.06–1.67)**	**0.014**
Sex								
Females	77/85/26	219/214/32	**1.38 (1.06–1.80)**	**0.017**	89/80/26	251/186/28	**1.47 (1.13–1.90)**	**0.004**
Males	109/109/25	269/209/41	**1.31 (1.03–1.66)**	**0.028**	116/108/25	256/216/47	1.07 (0.85–1.36)	0.547
Smoking								
Never	107/118/33	309/285/47	**1.34 (1.07–1.67)**	**0.011**	122/114/29	333/266/42	**1.29 (1.03–1.61)**	**0.026**
Ever	79/76/18	179/138/26	1.30 (0.97–1.75)	0.076	83/74/22	174/136/33	1.15 (0.87–1.52)	0.317
Drinking								
Never	104/116/33	321/308/51	**1.32 (1.06–1.65)**	**0.015**	124/106/30	355/278/47	**1.25 (1.00–1.56)**	**0.047**
Ever	82/78/18	167/115/22	**1.36 (1.01–1.82)**	**0.041**	81/82/21	152/124/28	1.19 (0.90–1.57)	0.223

[a]Derived from additive model using logistic regression analyses with an adjustment for age, sex, smoking and drinking status.

Supplementary Table 3: Heterogeneity test for rs217727 and rs2839701

Heterogeneity-test	rs217727	rs2839701
age		
p	0.803	0.312
I2(%)	0.0	2.1
sex		
p	0.775	0.076
I2(%)	0.0	68.3
smoke		
p	0.872	0.529
I2(%)	0.0	0.0
drink		
p	0.874	0.787
I2(%)	0.0	0.0

[a]for heterogeneity test based on χ^2-based *Q*-test.
